# A SYBR Green RT-PCR assay in single tube to detect human and bovine noroviruses and control for inhibition

**DOI:** 10.1186/1743-422X-5-94

**Published:** 2008-08-14

**Authors:** Alexandra Scipioni, Axel Mauroy, Dominique Ziant, Claude Saegerman, Etienne Thiry

**Affiliations:** 1 Department of Infectious and Parasitic Diseases, Virology, Faculty of Veterinary Medicine, University of Liege, Liege, Belgium; 2 Department of Infectious and Parasitic Diseases, Epidemiology and Risk Analysis Applied to Veterinary Sciences, Faculty of Veterinary Medicine, University of Liege, Liege, Belgium

## Abstract

**Background:**

Noroviruses are single-stranded RNA viruses belonging to the family *Caliciviridae*. They are a major cause of epidemic and sporadic gastroenteritis in humans and clinical signs and lesions of gastroenteritis were reported in bovines. Due to their genetic proximity, potential zoonotic transmission or animal reservoir can be hypothesized for noroviruses. RT-PCR has become the "gold standard" for the detection of noroviruses in faecal and environmental samples. With such samples, the control for inhibition of the reaction during amplification and detection is crucial to avoid false negative results, which might otherwise not be detected. The aim of the reported method is to detect, with a SYBR Green technology, a broad range of noroviruses with a control for inhibition.

**Results:**

A SYBR Green real-time RT-PCR assay was developed making use of a foreign internal RNA control added in the same tube. This assay is able to detect human and bovine noroviruses belonging to genogroups I, II and III and to distinguish between norovirus and internal control amplicons using melting curve analysis. A 10-fold dilution of samples appears to be the method of choice to remove inhibition. This assay was validated with human and bovine stool samples previously tested for norovirus by conventional RT-PCR.

**Conclusion:**

This SYBR Green real-time RT-PCR assay allows the detection of the most important human and bovine noroviruses in the same assay, and avoids false negative results making use of an internal control. Melting curves allow the discrimination between the internal control and norovirus amplicons. It gives preliminary information about the species of origin. The sensitivity of the developed assay is higher than conventional RT-PCR and a 10-fold dilution of samples showed a better efficiency and reproducibility to remove RT-PCR inhibition than addition of bovine serum albumin.

## Background

Norovirus is one of the four genera currently accepted into the family *Caliciviridae*. Other genera in this family include Sapovirus, which causes gastroenteritis in humans, as well as Lagovirus and Vesivirus, neither of which are pathogenic for humans. Noroviruses are small, non-enveloped viruses with a diameter of approximately 27–35 nm. They have a positive-sense, single stranded RNA genome [1]. Norwalk virus, the prototype strain of the genus norovirus, was first described in 1972 in association with an outbreak of gastroenteritis and vomiting involving children and staff at an elementary school in Norwalk, Ohio [2].

Noroviruses are now recognized as a common cause of human infectious gastroenteritis in all age groups, especially in restaurants and institutions such as nursing homes and hospitals [3-5]. They are one of the main causes of foodborne gastroenteritis [6,7]. Furthermore, several animal noroviruses genetically closely related to human noroviruses have been recently discovered [8-10]. Their existence raises important questions about animal reservoirs and potential zoonotic transmission [8]. The diagnostic of human and bovine noroviruses is impaired by the difficulties to replicate it in cell culture [11], although a tridimensional culture system was recently shown to be able to grow human noroviruses [12]. The full-length sequencing of different human norovirus genomes has allowed the development of reverse transcription polymerase chain reaction (RT-PCR) [13,14], which has become the gold standard for norovirus diagnosis [15]. Due to the genetic diversity among noroviruses, it is very difficult to find an appropriate primer pair that is both sensitive and specific for detection of all noroviruses. The most conserved region of the genome is the RNA polymerase gene and several primer pairs have been selected in that region [15], as the one used in this assay [16]. Real-time RT-PCR assays are more and more developed and has become the method of choice for the detection and the characterization of norovirus. Many different real-time RT-PCR assays for norovirus genogroups I and II had been developed [17-19] and co-detection of human and animal noroviruses was described in a multiplex assay [20] or simultaneously [21].

Noroviruses are usually detected in clinical specimens (faeces and vomit) and contaminated food, water or sewage [22-25]. Such samples commonly contain components reported to be (RT-)PCR inhibitors [26,27], leading to a high risk of false negative results or a decrease of the Ct value. A control to adequately detect problems with either RNase contamination or RT-PCR inhibitors is necessary to avoid false-negative responses for samples submitted for diagnosis [28,29]. An internal control is crucial to diagnostic (RT-)PCR assays. It is co-amplified with the target sequence and a negative result indicates a total (RT-)PCR failure. Also partial decrease of amplification capability can be estimated compared with the decrease of the internal control Ct value (internal control in the sample versus internal control alone).

The aim of this study was the development of a SYBR Green real time RT-PCR method able to detect the most important genogroups of noroviruses circulating in the human and bovine populations. This assay includes an internal RNA control and has been designed and validated for the diagnosis of noroviruses in human and bovine stool samples. Melting curve analysis allows the distinction between the internal control and norovirus amplicons and gives some indication about the species of origin. Moreover, the use of this single tube assay, cheaper than a TaqMan analysis, has the great advantages to detect (RT-)PCR inhibition that may lead to false negative results.

## Results

### Validation of the SYBR Green real-time RT-PCR assay – control of inhibition

The set up of the internal RNA control had been previously described [21] and the primers used in the SYBR Green real-time RT-PCR had been validated for their specificity by Vennema and collaborators [16].

Serial dilution of the norovirus internal RNA control transcribed *in vitro *demonstrated that 2 μl of the 10^9 ^fold dilution contained adequate template to produce a detectable product by melting curve analysis following real-time RT-PCR. This corresponds to a quantity of 1.9 × 10^-6 ^ng of internal RNA control or 5,800 copies [21].

**Figure 1:**
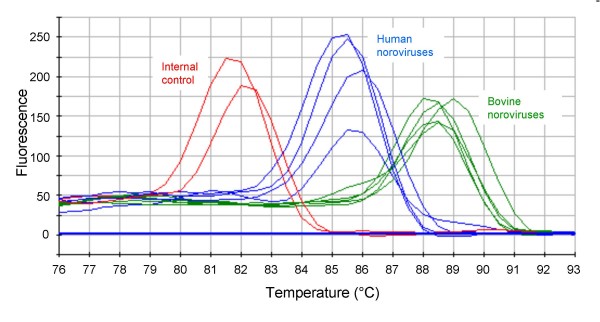
**Distinction between norovirus amplicons and internal control amplicon allowed by the melt curve analysis**. The internal control has a melting temperature around 81.5°C, human norovirus amplicons, around 85.5°C and bovine norovirus amplicons has a melting temperature around 88.5°C. Such differences in temperature are clearly visible on the curve.

Amplification of the internal RNA control produced amplicons with a melting temperature 3°C lower than norovirus amplicons (Figure 1).

**Figure 2:**
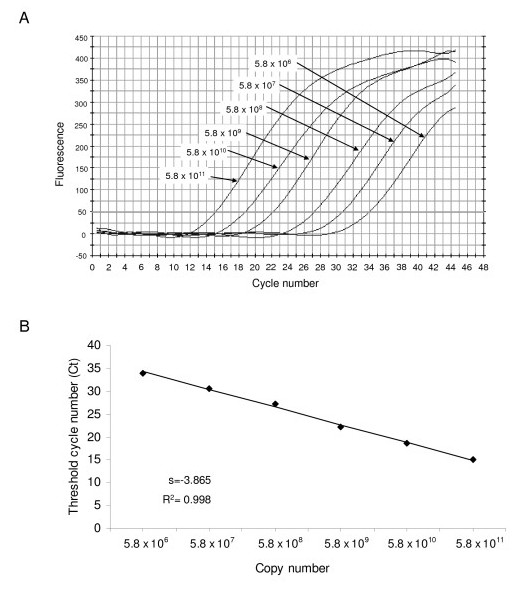
**Linearity of the SYBR Green assay**. (A) Detection of 10-fold serial dilution of a positive human sample by the SYBR Green assay performed from 5.8 × 10^6 ^to 5.8 × 10^11 ^molecules. (B) Standard curve of these dilutions, each dot representing the result of amplification for each quantity.

The exact amount of internal control to add in the mix with each RNA extraction from stool samples was determined using the detection limit of the internal control in real-time SYBR Green RT-PCR and checking the non-competitive amplification between the internal control and norovirus RNA. Different amounts of internal RNA control were added with 10-fold serial dilutions of extracted norovirus RNA. At the same time, a serial dilution of extracted norovirus RNA without internal control was tested with the SYBR Green real-time RT-PCR assay (data not shown). The quantity to add to a 25 μl mix was 3.8 × 10^-5^ng of internal RNA control, corresponding to 117,500 copies. The performances of the assay were evaluated using serially diluted internal RNA control (10-fold dilutions) from 5.8 × 10^6 ^to 5.8 × 10^11 ^copies and linearity was obtained (Figure 2).

**Figure 3:**
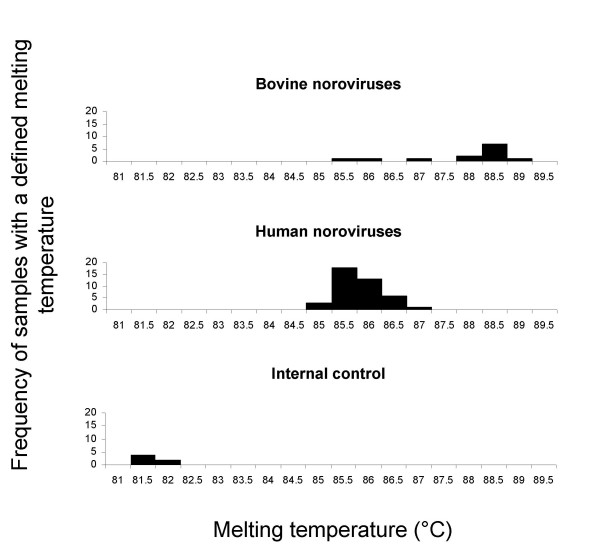
**Frequency occurrence of amplicon melting temperature for human noroviruses, bovine noroviruses, and the internal control**. The majority of bovine norovirus amplicons has a melting temperature around 88.5°C, about 3°C higher than the human ones. Though some bovine samples shown have a melting temperature similar to the human norovirus ones.

Most of the values obtained with the SYBR Green assay were within the 95% limits of agreement (mean of differences +/- 1.96 S.D. of the differences) showing satisfactory agreement (data not shown). For repeatability and reproducibility, the standard errors of measurement were less than 0.291°C and 0.354°C respectively. The mean melting temperatures were significantly lower for the internal control than both human and bovine noroviruses. Moreover the mean melting temperature for human noroviruses was significantly lower than the melting temperature for bovine noroviruses (Wilcoxon rank tests, P < 0.001) (Figure 3).

### Analysis of human and bovine stool samples

The comparison of the results obtained with the SYBR Green and the conventional RT-PCR assay is shown in table 1. In a first stage, the SYBR Green assay was performed on extracted RNA from stool samples. Three kinds of results were obtained: negative if a peak at 81.5°C was shown in the melting curve, positive if a peak around 85–88°C was shown and inhibition of reaction if there was an absence of these peaks (Figure 1). Samples containing human genogroup I or II noroviruses and bovine genogroup III noroviruses were tested and often showed different melting temperatures. The majority of bovine norovirus amplicons showed a melting temperature around 3°C higher than the human noroviruses (Figure 3).

**Table 1:** Comparison of the detection of human and bovine noroviruses by the conventional RT-PCR assay and the SYBR Green assay

		SYBR Green RT-PCR						
		+		-		Inhibition		

Conventional	+	44	**50**	0	**0**	6	**0**	50
RT-PCR	-	1	**4**	13	**32**	22	**0**	36

		45	**54**	13	**32**	28	**0**	86

Normal font: SYBR Green assay on RNA directly; **bold font**: SYBR Green assay modified to remove inhibition using 10-fold dilution of extracted RNA.

The use of the internal control for norovirus real-time RT-PCR diagnosis in 86 stool samples identified inhibition of RT-PCR in 32.6% of stool samples tested in this study (Table 1). Two different methods were used on extracted RNA from samples showing inhibition. One is a 10-fold dilution of the extracted RNA before testing with the SYBR Green assay and the second one is the addition of bovine serum albumin (BSA) in the mix. Among the 28 samples showing inhibition, a 10-fold dilution was active in all samples compared to BSA that failed to remove inhibition from 4 samples.

Considering the SYBR Green assay as the gold standard (Table 1), the relative sensitivity of the conventional RT-PCR used in this study was 92.6% (95% confidence interval (CI) 82.1–97.9). The relative specificity was 100% (CI 91–100) for all techniques. Confirming these results, Kappa value showed a high level of agreement between conventional RT-PCR and the SYBR Green real-time RT-PCR (Kappa value: 0.90; CI 0.81–0.99).

## Discussion

In this study we developed a sensitive and broadly reactive real-time SYBR Green RT-PCR assay that interestingly detects human genogroups I and II and bovine genogroup III noroviruses in a single tube, including an internal control. This assay takes into account that samples may contain inhibitors of PCR or RT-PCR [22,23]. The method uses one-step, hot start RT-PCR with thermostable DNA polymerase. The one step protocol simplifies the method and reduces the risk of contamination of RNA. Moreover it is useful for routine diagnosis as there is no post-amplification processing of the product.

The genetic diversity of noroviruses makes though to select a pair of primers capable of detecting all the different norovirus genogroups. The commonly used JV12-13 primer pair has been replaced by JV12Y-13I, which contains degenerated bases to allow the detection of a larger panel of noroviruses [16]. This primer pair is able to detect noroviruses belonging to human genogroups I and II and bovine noroviruses belonging to genogroup III [30]. The variability in the melting temperature between human and bovine noroviruses can be also explained by the genetic diversity among norovirus sequences [31,32], even if the amplicon is located in the polymerase region highly conserved among noroviruses. Therefore, this assay has the rare advantage to detect norovirus strains irrespectively of their origin (human or bovine). There are few methods described that allow the detection of both human and bovine noroviruses. Compared to the method recently published by Wolf and collaborators [20], our assay has the advantage to detect noroviruses in the same reaction tube and to control for inhibition of the reaction. It is also to detect mixed infection (presence of human and bovine noroviruses in the same sample), by the presence of two distinct peaks.

A common problem with RT-PCR is the presence of (RT)-PCR inhibitors which may cause false negative results. Therefore, to avoid such false negative results, the internal RNA control set up previously [21] was used in this real-time SYBR Green RT-PCR assay. It was synthesized *in vitro *from a foreign DNA template, in order to decrease interference with norovirus amplicons [33,34]. It has the advantages of representing no risk for human or animal health because it does not contain any infectious material, and being stable compared to live control viruses that can evolve and change during their replication. The norovirus amplification is favored compared to the internal control because the RT-PCR of the later results in a larger product. It is an essential property for its function. It means a decrease of the Ct value of the internal control if noroviruses are present in the sample.

With this internal RNA control, inhibition can be detected without the need of additional primer pairs or an additional reaction run for this purpose and the effective identification of samples containing endogenous inhibitors of RT-PCR is allowed. This improvement is crucial for early intervention and control in norovirus outbreaks.

With routine samples used for diagnosis, 36.8% of human samples and 24.1% of bovine samples showed presence of inhibition that can vary a lot among samples and may depend on the type of sample but also on the intrinsic characteristics of the sample (for example, herbivorous or omnivorous diet). When inhibition was detected in samples, the extracted RNA was tested a second time with the SYBR Green assay, on 10-fold diluted RNA or with BSA added in the RT-PCR mix. Sample dilution is often effective as the inhibitory factors can be diluted out, however, enough quantities of target nucleic acid must be present in order to be detected after dilution [35]. The addition of BSA, that is able to scavenge a variety of inhibitory substances [36], does not have this inconvenience. Our experience in using those two techniques to remove inhibitors led to the conclusion that a 10-fold dilution is more efficient and reproducible than the addition of BSA.

## Conclusion

In conclusion, the real-time assay described in this study is an accurate, sensitive, specific and quick method for the detection of a wide range of noroviruses belonging to genogroups I, II and III. At the same time it offers a method to detect samples containing inhibitors, avoiding false negative results by using an internal control. This assay will be applicable to clinical diagnosis in human and animal laboratories, detection of viruses in food or environmental samples. It is the first SYBR Green real-time assay that uses a single primer pair able to detect human and bovine noroviruses simultaneously. A 10-fold dilution of RNA appears to be the method of choice to remove inhibition.

The melting curve analysis gives presumption for the virus origin regarding the host and points out interesting samples to sequence for further studies (bovine norovirus with a melting temperature similar to the human norovirus ones). This property is of upmost importance regarding classification and study of transmission routes of noroviruses. This requires sequencing step in addition to detection. Although neither a zoonotic transmission, nor identification of an animal reservoir of norovirus have been already identified in natural condition, experimental evidence of cross infection was provided with successful inoculation of pigs with human norovirus [8,37]. Moreover bovine noroviruses have been detected in the food chain, in a bivalve mollusc sample which was contaminated with human noroviruses [38]. This increases the risk of crossing the species barrier and the probability of the emergence of recombinant viruses. In that context, a diagnostic assay that has the capacity to detect both human and bovine noroviruses is of high interest.

## Methods

### Human and animal stool specimens

Fifty seven human and 29 bovine stool samples were tested. Human samples were selected from faeces collected over a 2-year period (2000–2002) by the Medical Microbiological and Virological Laboratory of the University hospital of Liege and from outbreaks in Belgium provided in part by the Institute for Public Health in Brussels and the Virology Laboratory of the St Luc University hospital (2006–2007). Bovine samples were taken from faeces collected by the regional animal diagnostic laboratories "ARSIA" (*Association Régionale de Santé et d'Identification Animales*) in Belgium over a two year period (2002–2003). All bovine and human samples had been tested previously for norovirus by conventional one-step RT-PCR and sequenced for confirmation. The stool specimens were stored at -80°C. All positive samples were used in this study and 36 negative samples were randomly selected.

### Processing of stool samples, RNA extraction and conventional RT-PCR

The all procedure was already described [21]. Briefly, stool samples were 10-fold diluted in phosphate-buffered saline and RNA was extracted using the QIAamp viral RNA Mini Kit (Qiagen, Leusden, The Netherlands). A one-step RT-PCR kit, the Access RT-PCR System (Promega, Leiden, The Netherlands), was used with broadly reactive primer pairs, developed for the detection of noroviruses in stool specimens from humans or bovines [21].

### Real-time RT-PCR system

The real-time PCR assays were carried out on the iCycler (Biorad, Nazareth, Belgium) using iScript One-step RT-PCR kits for SYBR-Green assay (Biorad, Nazareth, Belgium) and used 2 μl of extracted RNA with 25 μl of master-mix with primers at 300 nM final concentration.

The primer set used was JV12Y-JV13I [16]. The quantity of 117,500 copies of the internal RNA control was added with each sample. The iCycler RT-PCR protocol included the following parameters: reverse transcription for 18 minutes at 48°C, 5 minutes at 95°C, followed by 45 cycles of 10 seconds at 95°C, 20 seconds at 48°C and 45 seconds at 60°C. Data were obtained during the elongation period. After the RT-PCR reaction, melting curve analysis was performed. To remove inhibition, BSA was added at a final concentration of 400 ng/μl in the RT-PCR mix or the extracted RNA was 10-fold diluted. A negative sample was added every 18 samples. All positive samples were confirmed by sequencing RT-PCR products.

### Statistical validation

Agreement of the mean melting temperature obtained with the real-time SYBR Green assay (repeatability, 35 duplicates and 10 triplicates, and reproducibility, 33 twice and 5 threefold) was measured according to a method described by Petrie and Watson [39].

Comparison between melting temperatures obtained with the real-time SYBR Green assay in each group (human noroviruses, bovine noroviruses and internal control) was performed using Wilcoxon rank tests and assuming unequal variance and data not distributed as a normal distribution [40].

All statistical analyses were carried out with Stata/SE [41]. Relative sensitivity and specificity were estimated with 95% confidence intervals assuming a binomial exact distribution. The limit of statistical significance of the conducted tests was defined as *P *≤ 0.05 and the Kappa coefficient was calculated.

## Competing interests

The authors declare that they have no competing interests.

## Authors' contributions

AS designed the internal control and the SYBR Green assay and did the real-time analyses. She drafted the manuscript. DZ carried out the conventional RT-PCR analyses of stool samples, and participated in analytical methods. AM was involved in the laboratory analyses and the draft of the manuscript. CS performed the statistical analysis. AS and ET conceived the study. ET is the head of the laboratory. All authors read and approved the final manuscript.
